# Anti-Tumor Necrosis Factor Alpha for Retinal Diseases: Current Knowledge and Future Concepts

**Published:** 2012-01

**Authors:** Alireza Mirshahi, René Hoehn, Katrin Lorenz, Christina Kramann, Holger Baatz

**Affiliations:** 1 Department of Ophthalmology, University Medical Center, Johannes Gutenberg-University Mainz, Mainz, Germany; 2 Aurelios Eye Center, Recklinghausen, Germany; 3 Department of Ophthalmology, J. W. Goethe University, Frankfurt / Main, Germany

**Keywords:** Tumor Necrosis Factor, TNF-α, Age-Related Macular Degeneration, Diabetic Macular Edema, Uveitis, Retinal Vein Occlusion

## Abstract

Tumor necrosis factor alpha (TNF-α) is a pro-inflammatory cytokine produced by macrophages and T-cells. It plays an important role both in inflammation and apoptosis. In the eye, TNF-α appears to have a role in the pathogenesis of inflammatory, edematous, neovascular and neurodegenerative disorders. Several TNF-blocking drugs have been developed and approved, and are in clinical use for inflammatory diseases such as rheumatoid arthritis, psoriasis and ankylosing spondylitis. TNF-α blockers are widely used in ophthalmology as an off-label alternative to “traditional” immunosuppressive and immune-modulatory treatments in noninfectious uveitis. Preliminary studies suggest a positive effect of intravenously administered TNF-α blockers, mainly infliximab, for treating refractory diabetic macular edema and neovascular age-related macular degeneration. Unfortunately, much of the current data raises considerable safety concerns for intravitreal use of TNF-α inhibitors, in particular, intraocular inflammatory responses have been reported after intravitreal injection of infliximab. Results of dose-finding studies and humanized antibody or antibody fragments (e.g. adalimumab) are anticipated in the coming years; these will shed light on potential benefits and risks of local and systemic TNF-α blockers used for treatment of diseases of the retina and choroid.

## INTRODUCTION

The tumor necrosis factor alpha (TNF-α) belongs to the group of pro-inflammatory cytokines produced by macrophages and T-cells and plays an important role in inflammation and apoptosis.^1^ Cytokines have a key function in establishing and sustaining acute and chronic inflammation. In several rheumatic diseases (e.g. rheumatoid arthritis, ankylosing spondylitis), TNF-α concentration is elevated in affected joints, which seems to play a prominent role in joint destruction. Similarly, overproduction of TNF-α has also been observed in Crohn’s disease and psoriasis arthritis.

In the eye, TNF-α appears to participate in the pathogenesis of inflammatory, edematous, neovascular, and neurodegenerative diseases.^2^ Injection of TNF-α into animal eyes induces breakdown of the blood–retina barrier.^3^ Furthermore, increased levels of TNF-α and TNF-receptors have been found in serum of humans with uveitis.^2^ Upregulation of TNF-α expression has been shown in keratocytes of patients with rheumatoid corneal ulcerations.^4^ Moreover, there is increasing evidence on TNF-α involvement in the pathogenesis of experimental retinal neovascularization, proliferative vitreoretinopathy, and macular edema.^5^–^7^ In an *in vivo* animal model of retinal injury, Berger et al showed that TNF-α plays a deleterious role in ischemia-reperfusion injury. Direct neutralization of this cytokine partially preserved retinal function.^8^ The diverse characteristics of TNF-α were attributed in part to the timing of its expression after injury. In their experimental study, Nagineti et al demonstrated that inflammatory cytokines, including interleukin 1 beta (IL-1β), interferon gamma (IFN-γ) and TNF-α, increase the secretion of vascular endothelial growth factor (VEGF) A and C by human retinal pigment epithelial (RPE) cells and choroidal fibroblasts, with VEGF being the most important factor for initiating pathological ocular neovascularization.^9^ TNF-α appears to play a major role in the pathogenesis of diabetic retinopathy in rats, and its pharmacological blocking leads to the inhibition of retinal cell death.^10^

In the 1990’s, pharmaceutical companies succeeded in developing drugs that neutralize TNF-α, i.e. TNF-α blockers. These agents were expected to have a positive effect on reducing symptoms of various diseases associated with increased TNF-α activity.^11^ The first TNF-α blockers were approved for therapy of rheumatoid arthritis in 1998. Several other TNF-α blockers have been developed and approved since then for other diseases, e.g. ankylosing spondylitis and psoriasis.

Currently available TNF-α blockers on the market include the following:

Infliximab (Remicade^®^), a chimeric human immunoglobulin G1 (IgG1) with a mouse variable fragment (Fv) having high TNF-α affinity and neutralizing capacity.

Adalimumab (Humira^®^), a fully humanized antibody against TNF-α.

Etanercept (Enbrel^®^), a recombinant dimeric protein generated by fusion of the ligand-binding portion of human type 2 TNF receptor (TNFR-2) linked to the fragment crystallizable (Fc) portion of human IgG1.

Certolizumab pegol (Cimzia^®^), a PEGylated humanized anti-TNF fragment antigen binding (Fab).

Golimumab (Simponi^®^), a human anti-TNF-α IgG1κ monoclonal antibody.

Since their introduction, ophthalmologists have used TNF-α blockers to treat several ocular diseases “off-label”. While most of the data now available addresses the treatment of uveitis, interesting new papers have focused on neovascular age-related macular degeneration, diabetic macular edema and retinal vein occlusions.

## SYSTEMIC ADMINISTRATION OF ANTI-TNF AGENTS FOR OCULAR DISEASES

The anti-inflammatory effects of TNF-α blockers make them an obvious therapeutic alternative for noninfectious ocular inflammation, especially when more conventional glucocorticoid or antimetabolite therapy has failed to control the inflammation, or their chronic use produces unwanted side effects. Treatment of any ocular disease with TNF-α blockers should be considered off-label, and it is advisable to obtain informed written consent.^12^, ^13^

Petropoulos et al reported 15 patients (28 eyes of 7 men and 8 women) with chronic, refractory, noninfectious uveitis receiving systemic anti-TNF-α therapy as single or adjunctive therapy.^14^ Ten patients had primary or secondary involvement of the posterior segment. All patients demonstrated improvement on fluorescein angiography (FA) and optical coherence tomography (OCT) as well as stable or improved visual acuity within the first two months after initiating anti-TNF-α therapy with infliximab (n=8) or adalimumab (n=2). No recurrence was observed and patients’ self-assessment of the effect of therapy was positive in all cases. Only one patient with retinal vasculitis associated with Crohn’s disease experienced serious side effects (arterial hypotension, fatigue, gastric symptoms).

In a comparative study of patients with retinal vasculitis due to Behçet’s disease, the infliximab treatment group (n=10) showed a significant decrease in inflammation, improvement in visual acuity and reduced ocular complications compared to the conventional therapy group (n=33).^15^ Moreover, the number of relapses was significantly less in the infliximab group than in the conventional group, i.e. 1.2 versus 6.3 relapses.

Diaz-Llopis et al presented outcomes of therapy in 19 patients with refractory uveitis including 33 eyes with cystoid macular edema (CME).^16^ After one year of treatment with adalimumab, 54.5% of eyes demonstrated complete resolution of CME and 31% had an improvement in visual acuity by 0.3 logMAR.

Systemic administration of infliximab (Remicade^®^) is by intravenous infusion and is usually started with a loading dose at initiation of treatment and two weeks thereafter. Maintenance treatment is administered at 4 to 6 week intervals. Inflammatory activity usually diminishes within 12 weeks after initiating treatment, otherwise such treatment should be reconsidered.

Adalimumab (Humira®) is administered by subcutaneous injections with single 40 mg doses at biweekly intervals. A loading dose may be given up to 160 mg (4 injections on the first day of treatment), when inflammatory disease activity must be quickly reduced. Should the response be insufficient, treatment may be increased to weekly injections.

Etanercept (Enbrel®) is usually injected subcutaneously at a 25 mg dose twice a week or as a single subcutaneous injection 50 mg per week.

Certolizumab pegol (Cimzia®) is available in preloaded syringes containing 200 mg for subcutaneous injection. A loading dose of 400 mg (i.e. two injections on the same day) is recommended at weeks 0, 2 and 4. Injections of 200 mg are administered biweekly thereafter.

Golimumab (Simponi®) is also available in preloaded syringes for subcutaneous injection. These syringes contain 50 mg of the agent and injections are given once monthly. In patients weighing over 100 kg and unresponsive to treatment after 3 to 4 months, the dosage may be increased to 100 mg (two syringes on a single day) once a month.

Since the immune system may produce antibodies against any administered TNF-α inhibitor, its therapeutic efficacy may diminish over time. When that happens, switching to another TNF-α blocker may be considered, however, the efficacy and side-effects of the second drug must be monitored meticulously.

### Complications and adverse reactions

The most severe side effects of TNF blockers are due to increased susceptibility to infectious diseases, most notably tuberculosis and other respiratory tract infections and gastrointestinal infections, e.g. hepatitis B reactivation.

Immune-mediated side effects may also occur such as atopic dermatitis, rash, hypersensitivity types I, II, III, lupus-like reactions and exacerbation of autoimmune diseases (e.g. multiple sclerosis). Other reported side effects include thromboembolic disorders, congestive heart failure, and malignancies, especially lymphomas and skin tumors.

### Systemic use of TNF blockers for diseases other than noninfectious uveitis

In 2005, Markomichelakis et al reported three cases of CNV regression secondary to age-related macular degeneration in patients under systemic administration of infliximab for inflammatory arthritis.^17^ The arthritis and neovascular age-related macular degeneration happened to coincide in these patients. An increase in visual acuity (VA) was observed in all three patients; improvement in one of them lasted for 18 months. In a recent prospective randomized study, Sfikakis and coworkers treated 11 patients with diabetic macular edema refractory to laser treatment with either intravenous infliximab (5 mg/kg) at weeks 0, 2, 6, and 14, followed by placebo at weeks 16, 18, 22, and 30, or vice-versa.^18^ VA improved significantly in 8 eyes of the immediate infliximab group, and this effect was sustained during the placebo phase. The authors suggest a larger trial to shed light on the efficacy of local or systemic TNF-blocking therapy in diabetic macular edema. Given that the treatment of refractory diabetic macular edema is challenging,^19^ results of this study may lead to the evolution of a new treatment modality for this sight-threatening disease.

These observations, together with the hypothesis that TNF-α may play a decisive pathogenetic role in the development of neovascularization in the eye, are leading to the development of animal models and human studies on intravitreal application of anti-TNF drugs in vascular and neovascular diseases of the retina and choroid.

## INTRAVITREAL INJECTION OF ANTI-TNF AGENTS FOR RETINAL DISEASES

Systemic TNF-α inhibitors are established drugs for therapy of rheumatic and auto-immune diseases, and positive effects have been observed in concurrent retinal and inflammatory eye diseases. The particular nature of ocular diseases, i.e. being restricted to ocular tissues, allows use of the vitreous cavity as a drug reservoir. Thus attempts to inject TNF-α inhibitors intravitreally (as routinely performed with anti-VEGF agents) is the next step. Anticipated advantages include bypassing the notorious side effects of systemic TNF-α inhibitors and achieving higher local concentrations.

Two current studies investigated the human vitreous for inflammatory mediators. Yoshimura et al collected vitreous specimens from 345 eyes with several retinal diseases such as diabetic retinopathy (DR), central retinal vein occlusion (CRVO), rhegmatogenous retinal detachment, and diabetic macular edema; they did not observe an elevated concentration of TNF-α in any of these disorders as compared to the control group (patients with idiopathic macular hole or macular pucker)^20^, but they did detect elevated concentrations of IL-6, IL-8 and monocyte chemoattractant protein-1 (MCP-1). Contradictory to their study, Suzuki et al analyzed the expression profile of cytokines in the vitreous fluid of 86 patients with DR (n=76) and CRVO (n=10), noting a significantly higher concentration of TNF-α in the vitreous of CRVO patients (234.9 pg/ml versus 158.11 pg/ml) 21. However, they also measured significantly higher concentrations of IL-8, IL-10, IL-13, immune-protein 10 (IP-10; interferon inducible protein-10), MCP-1, macrophage inflammatory protein-1 beta (MIP-1β) and VEGF in both conditions. Moreover, through a VEGF correlation analysis they found that anti-inflammatory cytokines such as IL-10 and IL-13 may be involved in the pathogenesis of DR and CRVO. These studies do not support the hypothesis that TNF-α plays a major role in primary non-inflammatory retinal diseases, however it may be that the choroid or retina themselves are the locus of increased TNF-α concentration, not the vitreous body.

In light of these results, it is not surprising that results of intravitreally administered TNF-α inhibitors are so heterogeneous. Wu and coworkers performed an interventional, multicenter retrospective study on diabetic macular edema.^22^ They treated 39 eyes with intravitreal adalimumab 2 mg/0.1 ml (n=5), infliximab 1mg/0.05 ml (n=15) or infliximab 2mg/0.1 ml (n=19). All patients had received at least three injections of VEGF inhibitors before. VA did not increase significantly in either the adalimumab or the 1mg infliximab group after three months; in the 2 mg infliximab group it even deteriorated. The authors also investigated macular thickness, which decreased in both infliximab groups and remained unchanged in the adalimumab group. Local side effects occurred only in the 2 mg infliximab group, with 42% developing severe uveitis that resolved under topical steroids or after vitrectomy. No systemic side effects were noticed.

Giganti et al treated two patients with neovascular AMD and two patients with diabetic macular edema with low-dose intravitreal infliximab (0.5 mg/0.05 ml).^23^ Pre-treatment of all four patients included anti-VEGF therapy and/or laser coagulation and/or photodynamic therapy. Only one patient with CNV and chronic CME secondary to AMD achieved better VA and remained without inflammatory side effects. The three other patients (one received two injections) showed a decrease in VA and developed vitritis or panuveitis. After six months, all patients demonstrated decreased responses on standard electroretinography and microperimetry. Three patients tested positive for human antichimeric antibodies. The authors deduced that low-dose infliximab is immunogenic, probably retinotoxic, and not well tolerated.

There is only one report by Theodossiadis and coworkers describing three patients demonstrating improved VA after intravitreal infliximab for neovascular AMD. Their first patient had already received three injections of ranibizumab before undergoing an injection of 1 mg infliximab; VA improved from 20/200 to 20/100.^24^ Macular edema initially decreased by 20%, followed by recurrence and vision loss after 8 weeks. A second injection with 2 mg infliximab led to complete regression of intra- and subretinal fluid in conjunction with an improvement in vision to 20/40. After seven months, the edema recurred and VA dropped to 20/100. Their second patient received two injections of 2 mg infliximab two months apart after two previous ranibizumab injections. Vision improved from 20/200 to 20/70 with a consecutive reduction in macular thickness. The last visit was two months after the second injection. The only observed side effect in this patient after the second injection was “vitreous opacities without inflammation”, which the authors attributed to a mild vitreous hemorrhage. Their third patient, pre-treated with one ranibizumab injection, also received two infliximab 2 mg injections, 3 months apart. VA increased from 20/100 to 20/30 at the last visit, two months after the second injection. No side effects were reported in patients one and three.

Current knowledge suggests that intravitreal TNF-α inhibitors have no permanent effect on macular edema in neovascular AMD or diabetic maculopathy. The three aforementioned patients showing visual improvement had short follow-up of only two months and markedly less pre-treatment than other patients and subjects studied by Wu et al. TNF-α inhibitors have been mainly used in chronic, long-lasting refractory disease where visual improvement is less likely. Dosage seems to be critical. A “safe” dosage of infliximab might be 1 mg/0.05 ml, as no inflammatory reactions were reported using this concentration, while lower or higher concentrations lead to frequent severe inflammatory reactions. Adalimumab 2 mg was well tolerated. Despite local application, systemic antibodies against non-human parts of the inhibitors have been detected, compromising treatment success. Thus, fully-humanized antibodies may solve this crucial problem.

Current data do not support the routine use of intravitreal TNF-α inhibitors in any retinal disease outside controlled trials. Further investigations, particularly dose-finding studies, will shed light on the potential therapeutic benefits of intravitreal or systemic anti-TNF therapy for vascular and neovascular diseases of the choroid and retina. We await the results of intravitreally administered humanized antibodies such as adalimumab in near future.

## SUMMARY AND CONCLUSION

Although the systemic use of TNF-α inhibitors is an appropriate off-label alternative to “traditional” immunosuppressive and immune-modulatory treatments in noninfectious uveitis, its use in vascular and neovascular retinal diseases has not yet been thoroughly examined. While preliminary results for intravitreal treatment of neovascular AMD were promising, subsequent case reports have raised considerable safety concerns, particularly the occurrence of intraocular inflammatory responses after intravitreal injection of infliximab. Dose-finding studies and the use of humanized antibodies are probably crucial for the success of intravitreal application. Further investigations will determine the role of TNF-α blockers in treating diseases of the choroid, retina and macula.

## METHOD OF LITERATURE SEARCH

Literature selection for this review was based on a MEDLINE database search in June 2010 and again in June 2011 using the terms tumor necrosis factor, TNF, retinal disease, macular disease, intravitreal injection, uveitis. All publication years were searched, and relevant articles related to intravitreal injections of anti-TNF substances were included. With regards to systemic use of anti-TNF drugs selected articles were included for this manuscript. Articles in foreign languages with abstracts in English were included as well.
